# Pattern of domestic violence amongst non-fatal deliberate self-harm attempters: A study from primary care of West Bengal

**DOI:** 10.4103/0019-5545.49448

**Published:** 2009

**Authors:** Arabinda N. Chowdhury, Arabinda Brahma, S. Banerjee, M. K. Biswas

**Affiliations:** Institute of Psychiatry, Kolkata, West Bengal, India

**Keywords:** Deliberate self-harm, domestic violence, pesticide poisoning, primary care, Sundarban

## Abstract

**Objective:**

Evaluation of various clinico-demographic variables and pattern of domestic violence in non-fatal deliberate self-harm (DSH) attempters admitted in 3 Block Primary Health Centers (BPHC) of Sundarban region of West Bengal, India in the year 2002.

**Materials and Methods:**

A prospective study of 89 DSH cases admitted at 3 Sundarban BPHCs by using a specially designed DSH register and a questionnaire on domestic violence in Bengali along with detail clinical interview.

**Results:**

Among the total of 89 DSH cases (23 male and 66 female), young (less than 30 years), female sex, low education and married status constituted major part of the sample. Pesticide poisoning was the commonest mode of DSH attempt. Typical stressors found were marital conflict or conflict with in-laws or guardian. A majority of DSH attempters (69.6%) experienced more than one form of domestic violence. Poverty and unemployment in the family were strongly associated with domestic violence. Among female DSH attempters, the most common perpetrator was husband (48.48%) followed by in-laws (16.67%) and parent (34.78%) was the most common perpetrator among males.

**Conclusion:**

Both DSH and domestic violence are serious socio-clinical issue of a major public health concern in the Sundarban region. Stressful life situations and various types of victimizations in the family intermixed with easy availability of lethal pesticides in this agriculture dependent community may facilitate the impulse of self-harm behavior, especially among the young housewives. Timely psychosocial intervention through community psychiatry program may mitigate the impact of psycho-cultural stressors and thus may help to reduce the morbidity and mortality from DSH.

## INTRODUCTION

Suicidal behavior and pesticide ingestion is a growing public health challenge in all the agricultural societies. Presently both fatal and non-fatal DSH are major public health concern globally. Fatal DSH (i.e. suicidal death) is accounting for more than 900,000 loss of life in 1995.[1] The rate of non-fatal DSH is 10 times more than the fatal DSH.[2] In India, the current national suicide rate is approximately 10.3 per 100000 populations.[3] The reasons for self-harm behavior may vary with cultures and societies.[4]

Various researches on suicidal behavior revealed that young age, female sex, low educational level and married persons are at potential risk for both fatal and non-fatal DSH.[5–7] Some Indian studies on fatal DSH found that only 3.4% of the patients were suffering from designated mental illnesses, and thus highlighted the importance of social factors in DSH decisions.[8] The relationship between self-harm behavior and domestic violence is an emerging research issue of much debate and unfortunately no convincing data is available till date to comment precisely on the relationship between DSH and domestic violence.

Domestic violence can range from verbal or psychological abuse including intimidation, harassment, damage to property, threats to kill or to harm, restraints of normal activities or freedom and denial of access to resources to physical abuse and sexual assault or rape.[9] More often than men, women are targets of violence. Although reliable data on the prevalence of violence against women by their close partners and thereby the chances of DSH are scarce, especially in developing countries like India, a growing body of research confirms its positive influences.[10–12]

The present prospective study aims to assess the clinico-demographic risk factors associated with non-fatal DSH with a special reference to identify pattern of domestic violence among the admitted self-harm attempters in the 3 BPHCs of Sundarban region of south 24 Parganas, West Bengal, India during the year 2002.

## MATERIALS AND METHODS

### Study area and period

**Figure 1:**
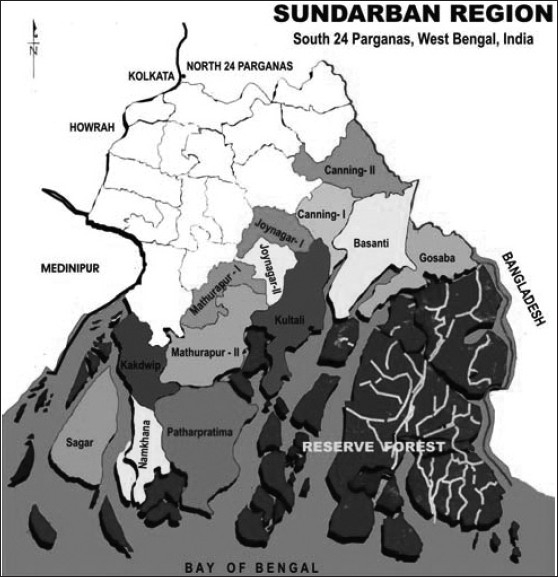
Sundarban under South 24 Parganas district (Not to scale)

Sundarban is the southernmost part of the state of West Bengal at the confluence of the river Hooghly to the Bay of Bengal. Sundarban is a socio-economically under-development region, with geographical inaccessibility, constantly changing landmasses and adverse climate conditions. Both the literacy rate and per capita income is much lower than the state average.[13] About 88.5% inhabitants are dependent on agriculture. Out of 13 blocks under South 24 Parganas district, Sagar is the most western, Namkhana is in the middle and the Gosaba is the most eastern island blocks of the Sundarban [Figure 1]. Each block has one BPHC. As a part of a DSH Prevention program in Sundarban region, the present work was conducted between Februarys to April 2002.

### Clinico-demographic assessments

Detail interview of DSH cases, who agreed, were conducted after taking ethical permission from the Block Medical Officer. As the total interview process was a lengthy one, it was divided into three sessions. A total of 51(4 died) male and 99 (3 died) female interviews were attempted but only 23 male and 66 female (a total of 89, all survived) interviews were completed. A DSH register (a 20-point DSH Case History sheet written in Bengali) was designed for the collection of socio-demographic data at first contact. Clinical findings of the cases were reported elsewhere. A Bengali questionnaire on domestic violence was used to assess: type of abuse, the relationship of the case to the abuser, stressful living conditions and the relationship of the incident to abuse.

## RESULTS

### Socio-demographic profile

The mean age of the 89 cases was 28.3 ± 13.8 years. Of them, 25.8% were males (Mean Age 30.5 ± 10.5 years) and 74.2% females (Mean Age 27.5 ±14.7 years). Most cases (74.1%) belonged to poorly educated groups (illiterate/can sign only/studied up to primary level). Females (81.8%) were significantly far lower in this aspect compared to males (52.1%) (Fisher's Exact Test, p=0.018*). Majority of the cases (78.7%) were married. Proportion of married cases was higher in females (78.8%) compared to males (78.3%). The observed difference was not significant (Fisher's Exact Test, p= 0.87, N.S.). Occupation wise, most females were housewives or doing household works (83.3%) and students (26.1 %) dominated among males and this difference was significant (Fisher's Exact Test, *p*=1.67 × 10^−9****^).

### Clinical profile

Use of poison was the most common DSH method in both sexes, with 100% females and 82.6% males using it [Table 1]. Hanging was seen among males only. The observed difference was significant (Fisher's Exact Test, p=0.004**). Gender wise distribution of the reasons for DSH shows that the conflict with spouse was by far the commonest cause in both the sexes (with slight female predominance) followed by conflict with parents (slightly more common in males) and this difference was significant (Fisher's Exact Test, p=0.033*.). ‘Other’ reasons include infertility, impotence; extra-marital affair of spouse, quarrel with other family member and political dispute etc.

**Table 1:** Methods and causes of DSH according to gender

Variables	Male		Female		Total	
			
	n=23	%	n=66	%	n=89	%
Methods of DSH						
Poisoning	19	82.6	66	100.0	85	95.5
Hanging	4	17.4			4	4.5
Causes of DSH						
Broken love affair			2	3.2	2	2.2
Conflict with parents	11	42.3	15	23.8	26	29.2
Conflict with in-laws			7	11.1	7	7.9
Chronic Illness	2	7.7	2	3.2	4	4.5
Dowry related	1	3.8			1	1.1
Economic distress	2	7.7			2	2.2
Failure in examination			1	1.6	1	1.1
Marital conflict	8	30.8	30	47.6	38	42.7
Other	2	7.7	6	9.5	8	9.0

### Profile of domestic violence

Table 2 shows pattern of different abuses according to gender. Abusive language and slang words were used frequently against both the sexes (with slight female predominance), but the difference was not significant (t = 0.27, N.S.). Beating as a mode of physical abuse was relatively higher among female cases (36.36%) than the male (4.35%). Suffocation was found only in females. The observed difference was not significant (t = 0.63, N.S.). The comparative of scores on pattern of psychological abuse revealed that humiliation was significantly more perceived in both the sexes with a slight female predominance (30.30% vs. 21.74% male), but the difference was not significant (t = 0.65, N.S.). Sexual coercion was reported significantly more in females (6.06%) than any other mode of sexual abuse (t = 2.04*, p<0.05). Perceived score of deprivation and neglect according to sex did not differ significantly among the cases (54.55% females vs. 43.48% males) of DSH (t = 0.07, N.S.).

**Table 2:** Pattern of various forms of abuse according to gender

Variables	Male		Female		Total	
			
	n=23	%	n=66	%	n=89	%
Verbal abuse						
Abuse/slang words	7	30.43	46	69.70	53	59.55
Defaming	6	26.09	9	13.64	15	16.85
Nil	10	43.48	11	16.67	21	23.60
Physical abuse						
Beating	1	4.35	24	36.36	25	28.09
Burn	1	4.35	3	4.55	4	4.49
Suffocation	-	-	2	3.03	2	2.25
Restraint	1	4.35	1	1.52	2	2.25
Nil	20	86.96	36	54.55	56	62.92
Psychological abuse						
Threat	4	17.39	18	27.27	22	24.72
Humiliation	5	21.74	20	30.30	25	28.09
Mock execution	1	4.35	4	6.06	5	5.62
Nil	13	56.52	24	36.36	37	41.57
Sexual abuse						
Harassment	-	-	1	1.52	1	1.12
Coercion	-	-	4	6.06	4	4.49
Stalking	-	-	2	3.03	2	2.25
Incest	-	-	3	4.55	3	3.37
Rape	-	-	2	3.03	2	2.25
Nil	23	100	54	81.82	77	86.52
Deprivation and neglect						
Yes	10	43.48	36	54.55	46	51.69
No	13	56.52	30	45.45	43	48.31

Table 3 shows the gender-wise breakup of stressful living conditions amongst the DSH cases. Only 34.83% cases reported abuse of various substances (including alcohol) in the family, though the difference was not significant (t = 0.93, N.S.). Though about 60% of cases reported unemployment in the family with a slight male predominance (69.6% vs. 56.1% female), but this difference was not significant (t = 0.64, N.S.). Though most of the cases (73.03%) belong to lower socio-economic status depending on the net family income with a slight female predominance (74.24%) over male (69.57%), however the difference was not significant (t = 1.08, N.S.). Significantly majority of the females (78.79%) who reported domestic violence were living at their husband's house (t = 7.3*, (p<0.001).

**Table 3:** Gender-wise breakup of stressful living conditions

Perpetrator	Male		Female		Total	
			
	n=23	%	n=66	%	n=89	%
Spouse	4	17.39	32	48.48	36	40.45
In-laws	1	4.35	11	16.67	12	13.48
Parents	8	34.78	11	16.67	19	21.35
Siblings	1	4.35	1	1.52	2	2.25
Children	-	-	3	4.55	3	3.37
Others	-	-	3	4.55	3	3.37
Nil	9	39.13	5	7.58	14	15.73

Table 4 shows the gender-wise breakup of the perpetrator of domestic violence. Among females, husband (48.48%) was the commonest perpetrator followed by in-laws and parents. Parent (34.78%) was the most common perpetrator among males. 17.39% of males were reported abused by their wives, which showed no significance (t = 1.11, N.S.).

**Table 4:** Gender-wise breakup of perpetrator of domestic violence

Variables	Male		Female		Total	
			
	n=23	%	n=66	%	n=89	%
Substance abuse						
Yes	5	21.74	26	39.39	31	34.83
No	18	78.26	40	60.61	58	65.17
Unemployment						
Yes	16	69.57	37	56.06	53	59.55
No	7	30.43	29	43.94	36	40.45
Socio-economic status						
Lower	16	69.57	49	74.24	65	73.03
Middle	4	17.39	15	22.73	19	21.35
Upper	3	13.04	2	3.03	5	5.62
Present living place						
In-law's house	1	4.35	52	78.79	53	59.55
Father's house	22	95.65	14	21.21	36	40.45

## DISCUSSION

The present research at the primary care setting of Sundarban region reveals some interesting findings about the socio-demographic variables and pattern of domestic violence related to self- harm behaviors.

### Socio-demographic characteristics

Present study reveals that the self-harm behavior is most frequent in younger individuals (below 30 years of age) with a slight female preponderance and having low educational attainment, which corroborates with the earlier studies.[14–17] This study also endorses the previous research findings that the housewives and students are at high risk of attempting DSH.[7] and DSH attempt was found more in married individuals (more common in females), similar to other Indian studies.[6,14]

### Clinical characteristics

Poisoning was the most common mode (95.5%) of DSH irrespective of gender found in this study. Similar findings were reported in a number of Indian studies.[16,18] Marital disharmony and family conflict were found to be the most significant cause for self-harm attempt in the present sample. The research experience from different Focus Group discussion (FGD) with housewives in Sundarban region evinced the stressful life of married women as like that of most Indian villages. The authors believe that easy availability and accessibility of different types of lethal pesticides in the households may enhance the psychodynamic vulnerability for DSH among the married females[19] in this region.

### Domestic violence characteristics

Domestic violence is an all-pervasive, serious social malady with major public health implications. It has physical and psychological damaging effects and puts the person at increased risk of depression, DSH attempts, physical disorders including various psychosomatic dysfunctions.[20] In the present study, 12.4% of cases reported that they experienced at least one form of domestic violence. The majority (69.6%) experienced more than one form of violence, while 18% did not report any kind of family violence. All – verbal, physical, psychological and sexual abuse – were more common amongst female DSH attempters. Only women reported experience of sexual abuse, though the number (18.2%) was much less than other forms of violence. Sexual coercion (6.06%) was the most common mode of sexual abuse. Rape and incest were also reported by a few cases. This probably reflects underreporting of such cases because of strong association of stigma related to sexual assault in the society. It is noted that following a sexual abuse some women experience severe impairment, including depression[21] and social maladjustment.[22] The strong association between sexual abuse and DSH attempts in our respondents probably suggests that many had been unable to recover from these traumatic experiences, which is a potential area for preventive intervention. Deprivation of the basic needs of life, viz., food, clothing, shelter was also commonly associated with the study cases. In consistence with the general social tone of Sundarban rural community the women are ranked inferior, and they were deprived more of such basic needs than their male counterparts.

In the present study, the risk of DSH attempt was higher among cases that were poor, having more unemployed persons in the family and those who were less educated. Heise[23] suggested that poverty can increase the risk of violence in the family faced specially by women. These women are faced with enormous social, physical and economic stresses, which in association with the experience of domestic violence are likely to increase their vulnerability to mental morbidities including self-harm behaviors.[24] Studies have also shown that low academic achievement was one of the risk factors predicting physical violence by men.[25] Substance abuse (mainly alcohol) by the husband and harassment by the in-laws were other factors that were intimately associated with increased risk of DSH attempt, all of which have been well reported.[26] Alcohol has consistently considered as a risk marker for domestic violence, which is especially consistent across a range of settings.[27] Harassment by in-laws on issues related to dowry demands, which emerged as a risk factor for non-fatal DSH in this study, is particularly characteristic of the Indian setting. The dowry demand and related tortures has been in existence for many years. Despite efforts, this malpractice continues to survive and acts as a potential factor that has driven many women to attempt or commit DSH.[28]

Intimate partner violence affects between 25% and 54% of women in their lifetime.[29] A study from Pakistan showed that the commonest factors forcing women to commit DSH were facing domestic violence from husband and in-laws.[30] In this study, spouse (mainly husband) was found to be the commonest perpetrator of violence (40.5%), followed by in-laws and parents. In India, most of the societies are predominately patriarchal. Thus, the women who are assaulted by a close member of the family have no way out, because the system considers these acts of violence as applicable.[12] Furthermore, married women in Sundarban were exposed to varieties of other psychosocial stresses, ranging from hard physical labor (both domestic and in field), lack of adequate privacy and recreational facilities, to inadequate social support. The cumulative effects of these emotional stresses increase their vulnerability to DSH.[31]

### Study limitations

Certain limitations of the study need to be mentioned here. As DSH attempt and domestic violence are punishable offences and carry strong social stigma, detailed information collection about these acts were difficult. All assessments were based on self-report, and were likely to underestimate the true prevalence of domestic violence. Moreover, interference of the family members (probably because of the legal apprehension) was a big problem during the interview in some of the cases. Unwilling participation and incomplete interview were more with the female subjects. Therefore the total sample size of this study was small; hence the findings should not be generalized. The conclusions made should be interpreted as a pilot finding from a group of DSH attempters at the primary care level.

## CONCLUSION

Thus it can be concluded from the present study that younger age (less than 30 years), female sex, low educational level, married status, intra-familial conflicts (with spouse or parents) and domestic violence are the significant risk factors associated with self-harm behavior. Pesticide poisoning was the most common method of DSH and thus reflecting a positive association between impulsive suicidal behavior and easy availability of pesticides in the region. Public education against domestic violence and timely psychosocial management of the vulnerable cases through a protracted community based mental health program may help to reduce the frequency of DSH and domestic violence.
